# Learning-Based Visual Saliency Model for Detecting Diabetic Macular Edema in Retinal Image

**DOI:** 10.1155/2016/7496735

**Published:** 2016-01-14

**Authors:** Xiaochun Zou, Xinbo Zhao, Yongjia Yang, Na Li

**Affiliations:** ^1^School of Electronics and Information, Northwestern Polytechnical University, Xi'an, China; ^2^School of Computer Science, Northwestern Polytechnical University, Chang'an Campus, P.O. Box 886, Xi'an, Shaanxi 710129, China

## Abstract

This paper brings forth a learning-based visual saliency model method for detecting diagnostic diabetic macular edema (DME) regions of interest (RoIs) in retinal image. The method introduces the cognitive process of visual selection of relevant regions that arises during an ophthalmologist's image examination. To record the process, we collected eye-tracking data of 10 ophthalmologists on 100 images and used this database as training and testing examples. Based on analysis, two properties (Feature Property and Position Property) can be derived and combined by a simple intersection operation to obtain a saliency map. The Feature Property is implemented by support vector machine (SVM) technique using the diagnosis as supervisor; Position Property is implemented by statistical analysis of training samples. This technique is able to learn the preferences of ophthalmologist visual behavior while simultaneously considering feature uniqueness. The method was evaluated using three popular saliency model evaluation scores (AUC, EMD, and SS) and three quality measurements (classical sensitivity, specificity, and Youden's *J* statistic). The proposed method outperforms 8 state-of-the-art saliency models and 3 salient region detection approaches devised for natural images. Furthermore, our model successfully detects the DME RoIs in retinal image without sophisticated image processing such as region segmentation.

## 1. Introduction

Diabetes is a chronic disease that can cause many serious complications including diabetic retinopathy (DR, damage to the retina). DR is an important cause of blindness. One percent of global blindness can be attributed to DR [1]. Diabetic macular edema (DME) is the most common cause of visual loss in DR, which is due to leaking of fluid from blood vessels within the macula. Fortunately, the Early Treatment Diabetic Retinopathy Study (ETDRS) has been able to reduce moderate vision loss in patients with clinically significant macular edema (CSME) by approximately 50% [2]. Hence to prevent vision loss, early diagnosis of DME is very important. Because there are no visual symptoms in the early stages of DME, the retinal fundus images are recommended in the diagnosis and treatment. The fundus image analysis helps the ophthalmologists in understanding the onset and assessment of the diseases. A reliable determination of clinically meaningful regions of interest (RoIs) in retinal image is at the very base of strategies for DME diagnosis. The advent of new inexpensive fundus cameras and rapid growth in information technology has made the automated system for DME RoIs selection possible. Such a tool is going to be notably useful in health camps particularly, especially in rural areas in developing countries wherever an outsized population laid low with these diseases goes unknown.

Exudates are the single most important retinal lesion detectable in retinal images. However, hard and yellow exudates within 500 *μ*m of the center of the fovea with adjacent retinal thickening indicate the presence of clinically significant macular edema (CSME), as defined by ETDRS [2]. But automatic DME RoIs finding in retinal images is a very challenging task. Because other retinal features such as blood vessels and outside diameter (OD) of bulbus oculi also have the similar brightness patterns and gray level variations, the naive use of current low-level-RoI-extraction methods for retinal images would probably fail.

Nevertheless, the ophthalmologists are always capable of figuring out a very precise diagnosis. When they search for CSME in retinal image, attention helps them rapidly disregard the “usual” and find the “unusual” visual elements. Some computational models of attention have been proposed to predict where people look in natural scenes [3–5]. Though the existing saliency models do well qualitatively, the models have limited use in DME RoIs detection because they frequently do not match actual ophthalmologists' precise diagnosis (ground truth, GT).

In this paper, we propose three contributions to DME detecting. First, we introduce the computational visual saliency models in retinal images in the context of DME detection. Through this model, we emulate the ophthalmologists' first examination step where she/he defines and separates high informative DME diagnostic regions. Second, by analyzing the precise diagnosis, we choose only a few concepts that encompass comprehensive ophthalmologists' visual behaviors, clarify the interactions among them, and develop a method for implementing the visual saliency. The method combines the advantages of a low-level image characterization with a high discriminant power in terms of DME tissular and spatial properties, information learned from the ophthalmologists. Third, we show that our model which is able to detect the DME RoIs in retinal images outperforms the mainstream salient region detection schemes.

This paper is organized as follows.  Section 2 provides a brief description and discussion of some previous works.  Section 3 is devoted to description of the implementation of the model. In Section 3.1, we present the images, eye-tracking data, and ground truth data for saliency model research.  Section 3.2 describes the properties, derivations, and relationships of salience concepts. The detailed description of our model is in Section 3.3.  Section 4 evaluates our approach using three popular saliency model evaluation scores (AUC, EMD, and SS) and three quality measurements (classical sensitivity, specificity, and Youden's *J* statistic) with 8 state-of-the-art saliency models and 3 salient region detection approaches. The conclusions and perspectives are discussed in Section 5.

## 2. Related Work

The problem of automatically detecting DME RoIs in retinal image has been approached using many techniques [6]. Phillips et al. [7] have proposed a method for the quantification of diabetic maculopathy using fluorescein angiograms. A combination of shade correction and thresholding techniques was used for preprocessing. The exudates were then detected by thresholding which was calculated based on the distribution of gray levels in the image. Hunter et al. [8] used feature extraction and classification techniques for the automated diagnosis of referable maculopathy. The technique detects and filters the candidate points with strong local contrast. Segmentation of candidate regions was carried out next in order to find the location of lesions. The lesions were distinguished from nonlesions by feature extraction technique. Authors have used shape, color, and texture of the candidate and the contrast between it and the surrounding retinal background. A multilayer perceptron (MLP) was used as classifier which classifies the lesions as dark or bright. A two-stage methodology was used for the detection and classification of DME severity of fundus images [9]. The first step was a supervised learning approach by which the fundus images were classified as normal or abnormal. By examining the symmetry of macular region using a rotational asymmetry metric the abnormal fundus images were further classified into moderate and severe DME. Osareh et al. [10] used an automatic method for the classifications of the regions into exudates and nonexudates patches using a neural network. The fundus images were preprocessed using color normalization and contrast enhancement techniques. The images were segmented next into homogenous regions using fuzzy* C*-means clustering. Based on the location of the exudates at the macular region Lim et al. [11] have classified the fundus images into normal, stage 1, and stage 2 of DME. The exudates were extracted from the fundus images using a marker controlled watershed transformation.

A DME pathologic diagnosis is the result of a complex series of activities mastered by the ophthalmologists. Classical psychophysical theories suggest that complex visual tasks, such as ophthalmologist examination, involve high degrees of visual attention [12].

Today, many saliency models based on a variety of techniques with compelling performance exist. One of the most influential ones is a pure bottom-up attention model proposed by Itti et al. [3], based on the feature integration theory [13]. In this theory, an image is decomposed into low-level attributes such as color, intensity, and orientation. Based on the idea of decorrelation of neural responses, Diaz et al. [14] proposed an effective model of saliency known as Adaptive Whitening Saliency (AWS). Another class of models is based on probabilistic formulation. Torralba [4] proposed a Bayesian framework for visual search which is also applicable for saliency detection. Similarly, Zhang et al. [5] proposed SUN (Saliency Using Natural statistics) model in which bottom-up saliency emerges naturally as the self-information of visual features. Graph Based Visual Saliency (GBVS) [15] is another method based on graphical models. Machine learning approaches have also been used in modeling visual attention by learning models from recorded eye-fixations. For learning saliency, Kienzle et al. [16] and Tilke et al. [17] used image patches and a vector of several features at each pixel, respectively.

These computational models have been used to characterize RoIs in natural images, but their use in medical images has remained very limited. Jampani et al. [18] investigate the relevance of computational saliency models in medical images in the context of abnormality detection. Saliency maps were computed using three popular models: ITTI [3], GBVS [15], and SR [19]. Gutiérrez et al. [20] have developed a visual model for finding regions of interest in basal cell carcinoma images that has three main components: segmentation, saliency detection, and competition. The key insight from these studies is that saliency continues to play a predominant role in examining medical images.

## 3. Learning a Saliency Model for DME RoIs Detection

### 3.1. Database of Eye-Tracking Data

For learning the preferences of ophthalmologist visual behavior and recording their eye-tracking data, we established an eye-tracking database, called EDMERI database (eye-tracking database for detecting diabetic macular edema in retinal image). The EDMERI allows quantitative analysis of fixation points and provides ground truth data for saliency model research. Compared with several eye-tracking datasets that are publicly available, the main motivation of our new dataset is for detecting diabetic retinopathy region in retinal images.

**Figure 1:**
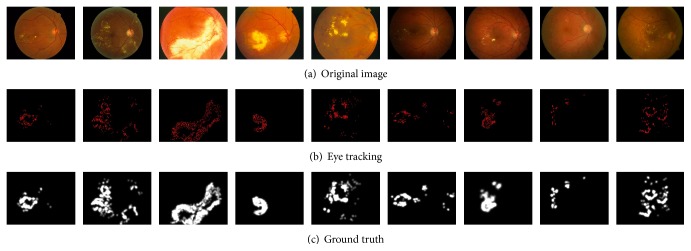
We collected eye-tracking data on 100 retinal images with diabetic macular edema from 10 ophthalmologists. Gaze tracking paths and fixation locations are recorded in (b). A continuous ground truth (b) is found by convolving a Gaussian over the fixation locations of all users.

The purpose of the current analysis was to model the cognitive process of visual selection of relevant regions that arises during detecting diabetic macular edema in retinal image. This reinforces our assumption that DME is a visible CSME feature. Under this constraint, we collected 100 images with DME (e.g., Figure 1(a)) from DIARETDB0 [6], DIARETDBI1 [6], MESSIDOR [21], and STARE [22], which are four standard diabetic retinopathy databases. These images stored in JPEG format were resized to 1152 × 1500 resolution. And we recorded eye-tracking data from ten expert ophthalmologists, with at least six years of experience, who were asked to view these images to find diabetic retinopathy regions. We used a Tobii TX300 Eye Tracker device to record eye movements at a sample rate of unique combination of 300 Hz. It has very high precision and accuracy and robust eye tracking and compensation for large head movements extends the possibilities for unobtrusive research of oculomotor functions and human behavior. A variety of researcher profiles, including ophthalmologists, can use the system without needing extensive training.

In the experiments, each image was presented for 10 s and followed by a rapid and automatic calibration procedure. To ensure high-quality tracking results, we checked camera calibration every 10 images. During first 1 s viewing, ophthalmologists maybe free viewed the histopathological image, so we discarded the first 1 s viewing tracking results of each ophthalmologist. In order to obtain a continuous ground truth of an image from the eye-tracking data of a user, we convolved a Gaussian filter across the user's fixation locations, similar to the “landscape map.” We overlapped the eye-tracking data collected from all ophthalmologists (e.g., Figure 1(b)) and then generated ground truth of the average locations (e.g., Figure 1(c)).

### 3.2. Relevant Properties and Bayesian Formulation

In this subsection, we will discuss the relevant properties of the visual saliency concepts in DME RoIs detection we have considered and the relationship among them. We assume that saliency values in a retinal image are relative to at least two properties, as described below.

*Feature Property (FP) (Saliency Is Relative to the Strength of Features in the Pixel).* In nature scene, features are traditionally separated into two types, high- and low-level features. High-level features include face, text, and events. Low-level features include intensity, color, regional contrast, and orientations. Since high-level features are more complex to define and extract in a retinal image, we only consider low-level features in this paper. For example, when the ophthalmologists are diagnosing the presence of DME, a pixel with strong yellow color feature tends to be more significant than one with weak ones.

*Position Property (PP) (Saliency Is Relative to the Location of the Pixel in the Image).* Actual ophthalmologists' precise diagnoses have shown that DME always appears within 500 *μ*m of the center of the fovea. That means the probability distribution of saliency for every pixel in a retinal image has a strong center bias property, so locational preferences in DME RoIs detection will be considered in our approach.

Considering the properties described above, the saliency of a pixel can be defined as the probability of saliency given the features and positions. Denote *F*_*X*_ = [*f*_*X*_^1^, *f*_*X*_^2^,…, *f*_*X*_^*n*^], *X* = [*x*, *y*] ∈ *I*, as a feature set including *n* features located in a pixel position *X* of image *I*. The saliency value can be denoted as *P*(*S*∣*X*, *F*_*X*_), where “*S* equal 1” indicates that this pixel *X* is salient (i.e., it is in DME RoIs) and zero otherwise. Based on the assumption that features can appear in all spatial locations, we assume *X* and *F*_*X*_ are independent of each other, as Zhang et al. [5] did. The probability of pixel *X* being salient can be written as

(1) PS ∣ X,FXPX,FX ∣ S·PSPX,FX=PX ∣ S·PFX ∣ S·PSPX·PFX=PS ∣ XPS·PS ∣ FXPS·PS=PS ∣ X·PS ∣ FXPS∝PS ∣ X·PS ∣ FX.

In (1), the term *P*(*X*∣*S*) is the probability of saliency given a position *X* and it corresponds to Position Property (PP). *P*(*S*∣*F*_*X*_) is the probability of saliency of the features appearing in location *X* and it corresponds to Feature Property (FP). As a result, the probability of saliency is clearly relative to two terms: PP and FP.

### 3.3. Learning-Based Saliency Model

**Figure 2:**
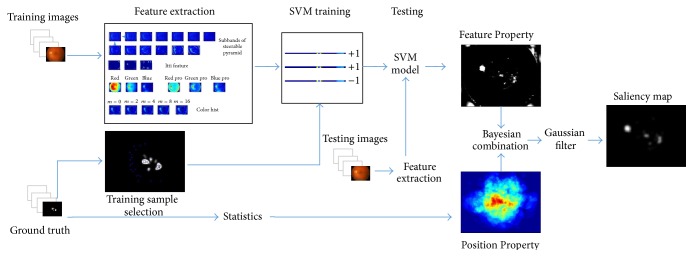
Illustration of learning process and saliency computing process. Feature Property: a set of low-level visual features are extracted from some training images. Feature vectors corresponding to the top 20% (bottom 50%) of the ophthalmologists' precise diagnoses (ground truth) are assigned +1(0) labels. Then a SVM classifier is trained from these features and is used for predicting DME on a test image. Position Property: we used a statistical analysis method to obtain it from ground truth. Finally, we combine Feature Property and Position Property adopting.

In contrast to manually designed measures of saliency, we follow a learning approach by using statistics and machine learning methods directly from eye-tracking data. Based on (1), when the ophthalmologists are diagnosing the presence of DME, there are two terms that affect saliency value in a pixel of a retinal image: FP and PP. Between these, FP can be learned from training samples using SVM; PP can be learned from ground truth of training images using statistical method. As shown in Figure 2, a set of low-level visual features are extracted from some training images. After the feature extraction process, the features of the top 20% (bottom 50%) points in the ground truth are selected as training samples in each training image. All of the training samples are sent to train a SVM model. Then, a test image can be decomposed into several feature maps and imported into SVM model to predict the FP. At the same time, PP also can be obtained from training images and their ground truth by statistical analysis. Finally, the two parts are combined, and a saliency map can be obtained after being convoluted with a Gaussian filter.

*Features Extraction*. After analyzing the DME dataset, we first extract a set of features for every pixel in each image including *m* × *n* pixels. Here, we use low-level features as they have already been shown to correlate with visual attention and have underlying biological plausibility [13, 23].

These features are listed below:

- Because they are physiologically plausible and have been shown to correlate with visual attention, we use the local energy of the steerable pyramid filters [24] as features. We currently find the pyramid subbands in four orientations and three scales, altogether thirteen images.
- Traditionally, intensity, orientation, and color have been used as important features for saliency, derivation over static images. We include the three channels corresponding to these image features as calculated by Itti's saliency method [12].
- We include three values of the red, green, and blue color channels as well as three features corresponding to probabilities of each of these color channels and five probabilities of each color as computed from 3D color histograms of the image filtered with a median filter at six different scales.

Eventually, all features are augmented in a 27D vector and are fed to classifiers explained in the next subsection. Each feature map is linearized into a 1 × *mn* vector (similarly for class labels).

*Feature Property*. In (1), FP is the relationship between a given feature set *F*_*X*_ appearing in position *X* and saliency value *S*. One of the simplest methods to determine saliency is to average all the feature values. However, some features may be more important than others, so giving the same weight to all features is not appropriate and will give poor results. Instead, we use SVM to implement Feature Property.

We compile a large training set by sampling images at diagnosis. Each sample contains features at one point along with a +1/−1 label. Positive samples are taken from the top *p* percent salient pixels of the precise diagnosis and negative samples are taken from the bottom *q* percent. We chose samples from the top 20% and bottom 50% in order to have samples that were strongly positive and strongly negative. We avoided samples on the boundary between the two. We did not choose any samples within 10 pixels of the boundary of image. We trained models using ratios of negative to positive samples ranging from 1 to 5 and detected no change in the resulting ROC curves, so we chose to use a ratio of 1 : 1. Training feature vectors were normalized to have zero mean and unit standard deviation and the same parameters were used to normalize test data. To evaluate our model, we followed the 10-fold cross validation method. The method partitions the database into ten subsets randomly, each with *M* images. Every subset is selected sequentially as a test set and the remainders serve as the training set. Each time we trained the model from 9 parts and tested it over the remaining part. Results are then averaged over all partitions.

We used the LIBLINEAR support vector machine [25], a publicly available MATLAB version of SVM, to implement FP. We adopted linear kernels as they are faster and perform as well as nonlinear polynomial and RBF kernels for our specific task. In testing, instead of predicted labels (i.e., +1/−1), we use the value of *W* ^*T*^ *f* + *b*, where *W* and *b* are learned parameters. We set the misclassification cost *c* at 1 and found that performance was the same for *c* = 1 to *c* = 10,000 and decreased when smaller than 1.

*Position Property*. As shown in (1), PP presents precise diagnosis preference for locations in an image. We implemented PP using a simple statistical method: sum up the values in the same position of density maps of database images, and normalize the result from zero to one. In the experiments, we denoted *M*_*i*_ as the *i*th ground truth image. Then the PP matrix *M*_*p*_ can be computed in

(2) $$\begin{array}{c} M_{p}=\frac{\sum_{i\in dataset}M_{i}-\min\left(\sum_{i\in dataset}M_{i}\right)}{\max\left(\sum_{i\in dataset}M_{i}\right)-\min\left(\sum_{i\in dataset}M_{i}\right)}. \end{array}$$

Based on the finding, the center prior in which the majority of fixations happen near the center of the image can be observed in *M*_*p*_.

*Property Combination*. The two matrices FP and PP, which are denoted as *M*_*f*_ and *M*_*p*_, respectively, are combined in the saliency map *M*_*s*_ by an intersection operation and a convolution operation, as shown in

(3) $$\begin{array}{c} M_{s}=\left(\;M_{f}•M_{p}\;\right)*GF. \end{array}$$

Here • denotes a Hadamard product and *∗* denotes convolution operation. We set the parameter of the Gaussian filter *δ* at 10 in the EDMERI database.

## 4. Experiment and Result

We validate our model by applying it to two problems: (1) DME RoIs prediction and (2) segmentation of the DME RoIs in a retinal image. We used EDMERI databases to evaluate our results; the size of each image was 1152 × 1500 pixels. We chose 100 training samples from each of the training images, for a total of 10,000 training samples. The database provided ophthalmologists' eye-tracking data as ground truth.

Since there is no consensus over a unique score for saliency model evaluation, we report results over three, including Area Under the ROC Curve (AUC), Earth Movers Distance (EMD), and Similarity Score (SS). A model that performs well should have good overall scores.

*AUC*. It is the most widely used metric for evaluating visual saliency. Using this score, the model's saliency map is treated as a binary classifier on every pixel in the image; pixels with larger saliency values than a threshold are classified as fixated while the rest of the pixels are classified as nonfixated [26]. Precise diagnoses are used as ground truth. By varying the threshold, the ROC curve is drawn as the false positive rate versus true positive rate, and the area under this curve indicates how well the saliency map predicts actual DME diagnoses. The two distributions are exactly equal when AUC is equal to 1, not relative when AUC is equal to 0.5, and negatively relative when AUC is equal to 0.

*EMD*. It represents the minimum cost of change of a distribution to another distribution. In this study, we use the fast implementation of EMD provided by Pele and Werman [27, 28]. EMD equal to zero means the two distributions are identical; a larger EMD means the two distributions are more different.

*SS*. It is another metric for measuring the similarities of two distributions. It first normalizes two distributions to let the sum equal one and then to sum the minimum values in each position. SS is always between zero and one. SS equal to one means two distributions are identical and SS equal to zero means two distributions are totally different.

Then, three quality measurements, classical sensitivity, specificity, and Youden's *J* statistic, were computed. The sensitivity and specificity were calculated for the whole set of classified pixels, that is, whether or not a pixel belonged to a RoI. Classically, the performance of a method is well described using sensitivity and specificity; they account for the individual result of hits or misses. However, we are interested in finding regions of interest, that is, collections of pixels with semantic meaning. Hence, the numbers of regions found by each method were also compared and the sensitivity of each method, regarding the number of RoIs, was also calculated.

*Sensitivity*. It is also called the true positive rate, or the recall in some fields, which measures the proportion of positives which are correctly identified, and is complementary to the false negative rate. The higher the sensitivity is, the more sensitive the diagnostic test is.

*Specificity*. It is also called the true negative rate, which measures the proportion of negatives which are correctly identified, and is complementary to the false positive rate. The higher the specificity is, the more precise the diagnostic test is.

*Youden's J Statistic*. It is also called Youden's index; this can be written as formula (4). Its value ranges from 0 to 1. The index gives equal weight to false positive and false negative values. The higher Youden's index is, the higher the authenticity the test has is. Consider

(4) $$\begin{array}{c} \text{Youden's index}=\text{sensitivity}+\text{specificity}-1. \end{array}$$

### 4.1. DME ROIs Prediction

#### 4.1.1. Analysis of AUC, EMD, and SS

As far as we know, this is the first investigation devoted to extraction of DME RoIs information from retinal images, using a bioinspired model. The developed method was compared with eight well-known techniques which had to deal with similar challenges, but in natural scene. We used them as the baseline because they also emulate the visual system, even though they are not specifically devised to detect relevancy in medical images; these eight models were AIM [29], AWS [14], Judd [17], ITTI [5], GBVS [15], SUN [5], STB [30], and Torralba [4]. We trained and tested our model over the dataset following 10-fold cross validation. For EDMERI, *M* = 10. The statistical results are shown in Table 1.

**Figure 3:**
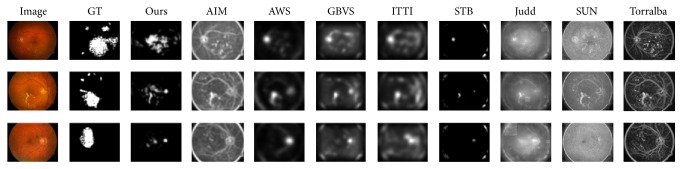
Some saliency maps produced by 9 different models from the EDMERI database along with predictions of several models using ROC. Each example shown by one row. From left to right: original image, ground truth, Ours, AIM, AWS, GBVS, ITTI, STB, Judd, SUN, and Torralba. It is obvious that Ours is more similar to the ground truth than other saliency maps.

Table 1 shows the comparison of evaluation performances of the 9 models in the EDMERI database. In this experiment, the average values of 10 times 10-fold cross validation in Table 1 are used for comparison. In the results, Ours has the best value in AUC, EMD, and SS. The AUC of our model is highest (0.8275), followed by Judd (0.7945). However, the average is only 0.6498. And the lowest value of EMD is shown in our model (8.1524), which is less than the average 12.8341. It means the results of Ours are more identical with ground truth than other models. Ours also has the best performance in SS with a value of 0.1930. The average value of SS is 0.0991, which just approximates half of Ours. Generally speaking, Ours has good performance in these three metrics. And Figure 3 presents some examples of the saliency maps produced by our approach and the other eight saliency models.

**Figure 4:**
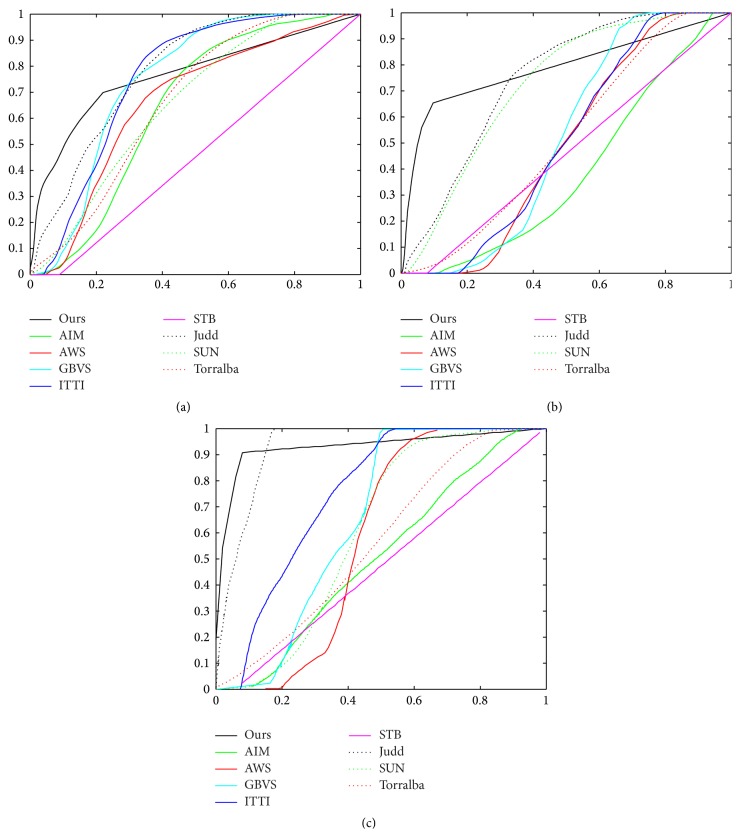
Some ROC curves produced by 9 different models from the three examples in Figure 3. ROC of Ours was higher than other models from the 5% to 20% salient region; GBVS, Judd, and ITTI2 got higher ROC when the salient region was larger than 60%. The salient DME RoIs in a retinal image region are generally under 20%. As a result, the performance of our method is better than other models when the salient region is small.

In Figure 4, we see the ROC curves of three examples in Figure 3 describing the performance of different saliency models. The size of the salient region plays an important role in the ROC method. Since it cannot separate salient regions from backgrounds using a certain threshold, the ROC method treats a saliency map as a binary classifier for ground truth under various thresholds. As shown in Figure 4, ROC of Ours was higher than other models from the 5% to 20% salient region; GBVS, Judd, and ITTI2 got higher ROC when the salient region was larger than 60%. This means that when the definition of the salient region changes, the rank of performance using the ROC method may change. However, the salient DME RoIs in a retinal image region are generally under 20% and even smaller in many cases. As a result, the performance of our method is better than other models when the salient region is small.

#### 4.1.2. Analysis of Sensitivity and Specificity

The ability of the different methods to extract DME RoIs information from retinal images was evaluated using conventional sensitivity and specificity measurements. These results are shown in Table 2.

Table 2 shows sensitivities and specificities of the 9 models in 50% salient region. Overall, all sensitivity, specificity, and Youden measurements evidence that our model outperforms the other models. The sensitivity of our model is 83.7%, which surpasses the average sensitivity 18.2%, followed by STB with 81.6% and GBVS with 80.1%. However, Judd had the lowest rate (only 37.2%), less than half of Ours. And the larger value of specificity (77.7%) is also shown in our model, which exceeds the average specificity 14.8%. Although the sensitivities of STB and GBVS are over 80%, their specificities are 74.1% and 57.0%, respectively; both are under Ours. The sensitivity of GBVS especially is even lower than the average. Owing to having the highest value of sensitivity and specificity, Youden's index (0.614) of our model is the highest among the 9 models, followed by STB with 0.557 and GBVS with 0.371. Average Youden's index is 0.356, which is only higher than half of Ours. The indisputable fact is that the higher Youden's index is, the higher the authenticity the test has is, and our model outperforms the other models in all sensitivity, specificity, and Youden measurements based on Table 2. Thus, Ours is suitable for extracting DME RoIs information from retinal images.

### 4.2. DME ROIs Detection

Almost all salient region detection approaches utilize a saliency operator, where from there they start to segment the most salient object. Because they are not specifically devised to detect relevancy in medical images, there is little study investigating the relevance of computational saliency models in medical images in the context of abnormality detection. Here, we used three well-known techniques as the baseline and showed that our approach could provide a good such starting point; these three models were ITTI [3], SR [19], and Achanta's [31].

**Figure 5:**
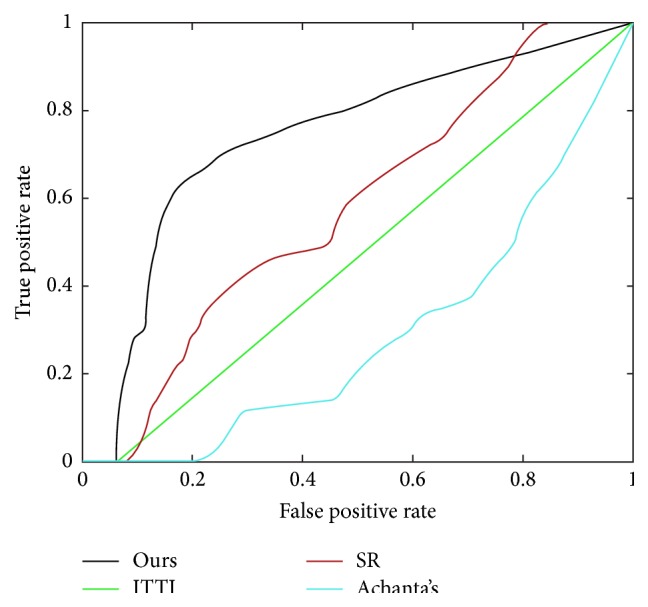
ROC curves for comparison of our model with ITTI, SR, and Achanta's.

**Figure 6:**
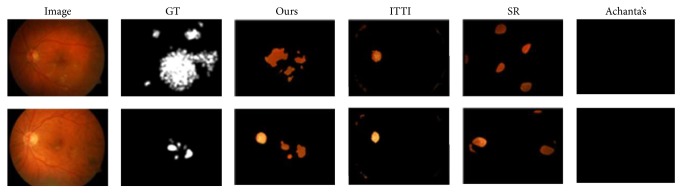
Some unnormalized saliency map for DME ROIs detection produced by 4 different models from the EDMERI database. Each example shown by one row. From left to right: original image, GT, Ours, ITTI, SR, and Achanta's. It is obvious that Ours is more similar to the ground truth than other saliency maps.

We calculate ROC curves in Figure 5 by binarizing the saliency map using every possible fixed threshold, similar to the fixed thresholding experiment in [31]. As seen from the comparison (Figure 5), our saliency model performs better while competing with the other three state-of-the-art models tailored for this task.  Figure 6 shows examples with diagnosis and detections of our model and the other three salient region detection models. As can be seen, our model is able to successfully detect the DME ROIs, ITTI's RoIs, and SR's RoIs mismatching the ground truth, and even worse, Achanta's cannot detect the DME ROIs.

## 5. Discussions and Conclusions

The present paper has introduced a novel strategy, a new visual saliency model using the Bayesian probability theory and machine learning techniques, for selecting DME RoIs in retinal images. The model is inspired in the first phase of a DME pathological examination, a process largely studied which starts by scanning the retinal images.

So far the underlying mechanism that controls a DME RoIs selection in retinal image has been poorly studied. Recent studies suggest that some visual mechanisms, such as the one that allows highlighting an object from the background and the visual attentional process, are connected. This fact suggests that the visual system is able to selectively focus on specific areas of the image, which besides are entailed with a high relevant meaning. Yet the idea is far from being fully exploited; our approach has been able to capture some basic facts; that is to say, that relevancy is a global property somehow constructed by integrating local features.

The proposed strategy is based on the interaction of Position Property and Feature Property and combined by a simple intersection operation using the Bayesian probability theory and machine learning techniques to obtain saliency maps. Our model is unlike traditional contrast-based bottom-up methods in that its learning mechanism has the ability to automatically learn the relationship between saliency and features. Moreover, unlike existing learning-based models that only consider the components of features themselves, our model simultaneously considers appearing frequency of features and the pixel location of features, which intuitively have a strong influence on saliency. As a result, our model can determine saliency regions and detect DME ROIs more precisely. Experimental results indicate that the proposed model has significantly better performance than other state-of-the-art models.

## Figures and Tables

**Table 1:** Performance comparison of nine models in the EDMERI dataset.

Metrics	GT	Ours	AIM	AWS	GBVS	ITTI	STB	Judd	SUN	Torralba	Average
AUC	1.0000	0.8275	0.5610	0.6081	0.6748	0.6419	0.4723	0.7945	0.6449	0.6235	0.6498
EMD	0.0000	8.1524	13.4767	13.2513	12.3116	12.8744	17.9996	12.3079	12.4320	12.701	12.8341
SS	1.0000	0.1930	0.0853	0.0987	0.1066	0.1044	0.0055	0.1033	0.0932	0.0988	0.0991

**Table 2:** Sensitivities and specificities of nine models.

	Ours	AIM	AWS	GBVS	ITTI	STB	Judd	SUN	Torralba	Average
Sensitivity (%)	83.7	50.7	57.0	80.1	69.6	81.6	37.2	65.6	63.7	65.5
Specificity (%)	77.7	59.2	64.5	57.0	58.1	74.1	60.1	60.0	55.4	62.9
Youden	0.614	0.099	0.215	0.371	0.277	0.557	−0.027	0.256	0.191	0.356
